# Possible shift in the ENSO-Indian monsoon rainfall relationship under future global warming

**DOI:** 10.1038/srep20145

**Published:** 2016-02-03

**Authors:** Sarita Azad, M. Rajeevan

**Affiliations:** 1 Indian Institute of Technology Mandi, Mandi 75001 Himachal Pradesh, India; 2 Indian Institute of Tropical Meteorology, Dr Homi Bhabha Road, Pashan 411 008 Pune, India

## Abstract

EI Nino-Southern Oscillation (ENSO) and Indian monsoon rainfall are known to have an inverse relationship, which we have observed in the rainfall spectrum exhibiting a spectral dip in 3–5 y period band. It is well documented that El Nino events are known to be associated with deficit rainfall. Our analysis reveals that this spectral dip (3–5 y) is likely to shift to shorter periods (2.5–3 y) in future, suggesting a possible shift in the relationship between ENSO and monsoon rainfall. Spectral analysis of future climate projections by 20 Coupled Model Intercomparison project 5 (CMIP5) models are employed in order to corroborate our findings. Change in spectral dip speculates early occurrence of drought events in future due to multiple factors of global warming.

The ENSO phenomenon is known to affect the inter-annual variability of the tropical climate system, including the Indian monsoon. As per the definition, El Nino refers to a situation where five consecutive three month moving average Oceanic Nino Index Average (ONIA) values exceed 0.5 °C. Analysis of observational data reveals that inverse relationship between ENSO events and Indian summer monsoon rainfall (ISMR) is statistically significant^1^,^2^,^3^,^4^,^5^ even though there is no one to one correspondence between them^6^,^7^,^8^. There are normal monsoon years associated with ENSO but most of the severe droughts in India are associated with the ENSO events. According to Saini and Gulati^9^ as per the defining conditions in terms of ONI, there have been 7 El Nino years (1980 onwards) of which five converted to Indian droughts. Therefore, seasonal prediction of ISMR is estimated based on its inverse relationship with ENSO^10^,^11^.

Observational temperature data for the past 100 years or so suggest an increasing trend in global mean temperature. The projections using the Intergovernmental Panel on Climate Change (IPCC) models suggest continuation of warming over the next 100 years, even though there is an uncertainty in the magnitude^12^. The ensuing global warming may have serious implications for the tropics, including changes in circulation and rainfall patterns over the monsoon regions. For example, global warming is likely to cause an increase in mean Indian monsoon rainfall^13^, accompanied by an increase in the frequency of extreme precipitation events^14^,^15^,^16^. A recent study by Cai *et al.*^17^ has reported that global warming will have a significant impact on ENSO behaviour by causing a pronounced eastward extension of the west Pacific warm front pool. Reorganization of atmospheric convention currents at such a massive scale will lead to enhanced frequency of extreme El Niño events almost doubling the occurrence over the next half of the 21^st^ century. As a result tropical precipitation would be adversely affected; this is credited to the projected surface warming over the eastern equatorial pacific which occurs faster than in the surrounding waters. This will facilitate rapid occurrence of atmospheric convection in the eastern equatorial region. However, previously some other studies have reported the weakening of ENSO-ISMR relationship^18^,^19^.

Variation in rainfall has a huge impact on the agricultural output in India. Both the extremes, floods and droughts, affect adversely food security, inflation and GDP of the country^20^. With the global warming, it is an important to examine how the ENSO-monsoon relationship changes in future climate. The present analysis is important exercise to understand the possible changes in predictability of monsoon rainfall based on the relationship with ENSO. Recently, it has been observed that the spectrum of Indian monsoon rainfall exhibits a dip in the 3–5 y period band^21^. It has been hypothesised that this phenomenon could be a consequence of the El Niño effect. In this context, several attempts have been made to describe the temporal variability of the ENSO-ISMR relationship on a 2–7 year timescale^22^. A spectral wavelet analysis revealed that the net effect of solar processes on rainfall operates in part indirectly through ENSO.

Under global warming scenarios, many studies have speculated the changes in ENSO and related precipitation variability^23^. In this regard, the present article aims to explore the ENSO-monsoon relationship with respect to global warming scenario RCP-8.5 24. To the best of our knowledge such studies have not been reported in the literature.

## Results

**Figure 1:**
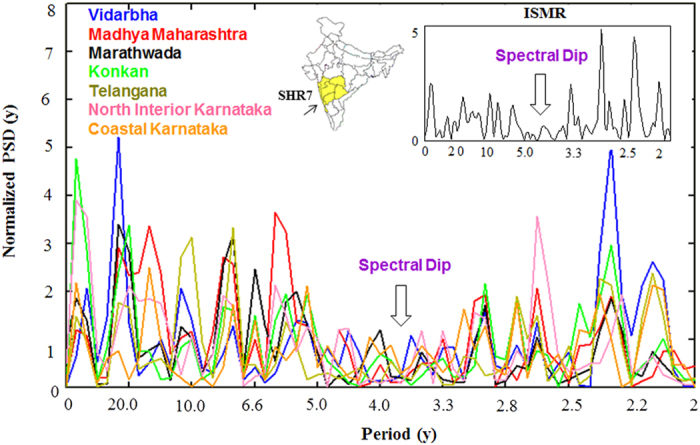
PSD of seven sub-divisional rainfall of SHR7 region and ISMR (inset) demonstrating spectral dip in the 3–5 y period band. India’s map is created using shape file which is available at survey of India website **www.surveyofindia.gov.in.**

**Figure 2:**
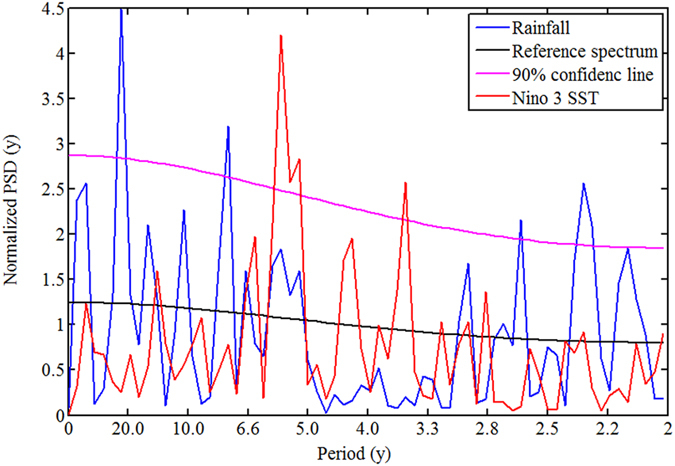
Inverse relation between ENSO-SHR7 rainfall in observed spectra during the time period 1871–2005.

The power spectral density (PSD) function of seven sub-divisions of a spectrally homogenous region (SHR7) rainfall for the time period 1871–2005 are estimated using Welch technique^25^,^26^ and shown in Fig. 1. It is clearly seen from the Fig. 1 that all the constituent sub-divisions show a spectral dip in the 3–5 y period band, centred around 4 y period. In our previous work, we have found that spectral peaks in the seven sub-divisions which constitute SHR7 are in near-coincidence with those from other sub-divisions, and such remarkable symmetry is not because of any statistical fluctuations^21^. This spectral dip is an indication of deficit rainfall and is also observed in ISMR (inset in Fig.1) and other homogenous regions (not shown in Fig.1). The main focus of this work is to show the inverse relation between ENSO and monsoon rainfall in the spectral domain. The Fig. 2 evidently depicts this relation, where it is shown that between 3–5 y period band, SHR7 rainfall shows low spectral density (defined as a spectral dip) whereas Nino 3 SST index shows significant high power density at 90% confidence level. To check if this inverse relation persists in future projections provided by CMIP5 data sets, firstly, we select models for rainfall in the historical time period (1871–2005) whose spectra match best with the observations using two statistical tests. These are defined as follows: (a) in order to confirm the existence of spectral dip in the model spectrum, we pose a criteria that there should not be any significant periods (above 90% confidence line) in the 3–5 y period band; (b) we define a null hypothesis that the mean of significant periods (above 90% confidence line) in the observed spectrum is same as model. This hypothesis is further accepted or rejected using Student’s t-test at 90% confidence level.

**Figure 3:**
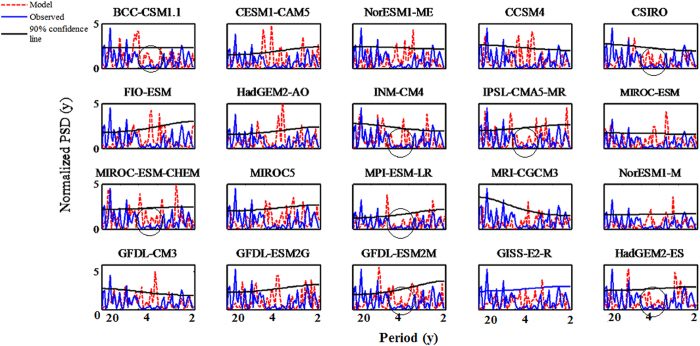
PSD of SHR7 rainfall from 20 CMIP5 models (red) against observations (blue). The eight models whose spectra match well with the observed are marked in circle.

**Figure 4:**
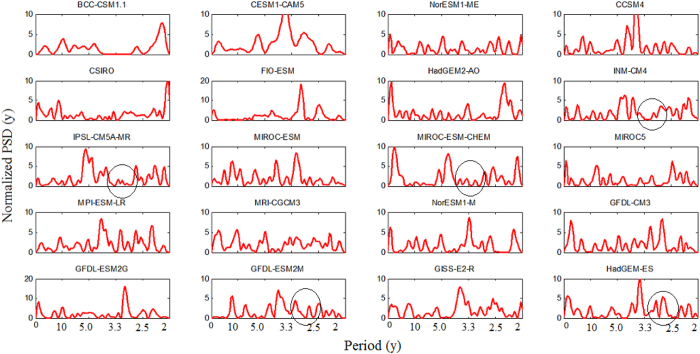
PSD of projected SHR7 rainfall from 20 CMIP5 models. The five models which show shift in the spectral dip are marked in circle.

**Figure 5:**
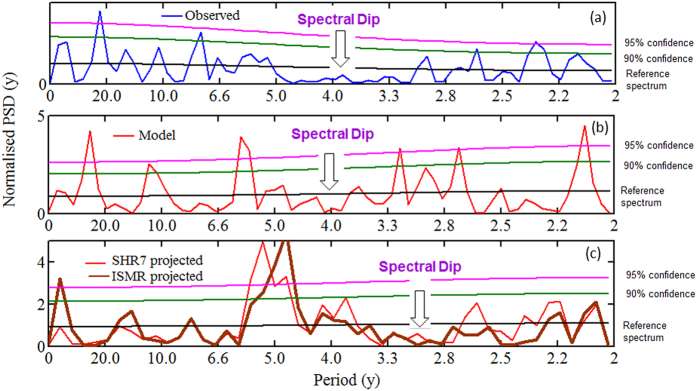
PSD of (a) observed; (b) model IPSL-CM5A; and (c) comparison of SHR7 rainfall and ISMR projected.

It is shown in Fig. 3 that the spectral dip which is present in the observed spectrum in the 3–5 y period band is captured by eight models, namely BCC.CSM1.1, CSIRO-Mk3.6.0, INM-CM4, IPSL-CM5A-MR, MIROC-ESM-CHEM, MPI-ESM-LR, GFDL-ESM-2M, HadGEM2-ES. In all of these eight models, there are no significant peaks above 90% confidence line with slight exception at the boundaries in INM-CM4, MIROC-ESM-CHEM, GFDL-ESM2M. We further perform the null hypothesis defined in (b). It is found that the null hypothesis is accepted for these eight models, i.e. mean of significant peaks (above 90% confidence level) in model spectra is same as observed. It is important to note that spectra of all the 20 models are not in corroborations with observations. However, some of these models are able to capture rainfall spectral contents upto some acceptable level which are used in this study. Similar concern has been reported on El Nino data^27^ where six models (out of 20 models) have shown superior performance and have been used for predicting the results. To examine the behaviour of the spectral dip in future, we estimate the PSD of projected rainfall of 20 models as shown in Fig. 4. It is seen from the figure that five out of the eight models mentioned above exhibit a shift in the spectral dip from 3–5 y period to 2.5–3.3 y period. Hence, projections reveal that the spectral dip which is centred at period 4 y in the historical data is likely to shift to 3 y period. It is interesting to note that 5 y periods which is not significant in the historic data is found to be significant in the projections of INM-CM4, IPSL-CM5A-MR, and MIROC-ESM-CHEM. Figure 5 demonstrates these two properties more explicitly. It is seen from the figure that the 5 y period which comes under spectral dip in the historic period of observations (Fig. 5(a)) as well as model (Fig. 5(b)) is 95% significant in the projection of model IPSL-CM5A-MR (Fig. 5(c)). Also, the spectral dip is shifted towards shorter periods, centred at 3 y period, and no periods are found to be significant in this dip (Fig. 5(c)). It is to note that this shift is observed in both SHR7 and ISMR rainfall. We wish to verify it further by analysing PSD of seven sub-divisions of SHR7.

As mentioned earlier, although spectral dip is present in ISMR and other homogenous Indian monsoon regions, we have selected SHR7 as a prime region for our analysis as it contains seven observations unlike single observation of ISMR. Therefore to confirm this shift in future, we estimate the PSD of seven sub-divisions of SHR7 and shown in Supplementary Fig. S1 (See the Supplementary Information) for the historic period 1871–2005 and for the projected period 2006–2100 is shown in Supplementary Fig. S2. To assess the similarities in the seven spectra of observed and model we check the two criteria as before. Particularly we examine the seven sub-divisions of eight models selected in previous section, namely, BCC.CSM1.1, CSIRO-Mk3.6.0, INM-CM4, IPSL-CM5A-MR, MIROC-ESM-CHEM, MPI-ESM-LR, GFDL-ESM-2M, and HadGEM2-ES and we found that the null hypothesis is accepted for these models. Particularly, the seven sub-divisional spectra of model IPSL-CM5A-MR are shown in Supplementary Fig. S3. It is interesting to note that the seven spectra in this model coincide well with other sub-divisions as witnessed in observations. This confirms the reliability of model performance as we have mentioned in our previous work^21^ that such spectral symmetry cannot be a result of any statistical fluctuations. Also outputs of this model show a shift in the spectral dip and 5 y period is significant in the projected data.

**Figure 6:**
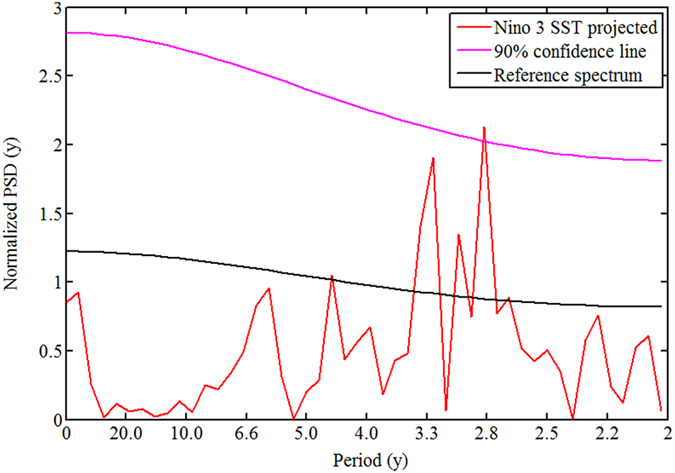
Projected changes in Nino 3 SST by model GFDL-CM3.

In order to further understand the inverse relation between ENSO and Indian monsoon rainfall, the similar analysis is performed on Nino 3 SST model simulations. We aim to find the models which are able to capture the high power density in the 3–5 y period band. These eight models are, CCSM4, CISRO, INMCM4, MIROC, MRI-CGCM3, GFDL-CM3, GFDL-ESM-2M and GISS-E2. Out of these eight models, it is found that four models exhibit a shift of spectral density toward shorter periods as seen in the case of rainfall. These four models are, CISRO, MRI-CGCM3, GFDL-CM3, and GFDL-ESM-2M. Figure 6 demonstrates this shift for model GFDL-CM3. The similar power spectral density approach has been employed recently to estimate the PSD of ENSO^28^. The most interesting result of our study is that the inverse relation between ENSO-monsoon rainfall is likely to persist in future as shown in Figures 5 and 6 for rainfall and ENSO, respectively.

## Discussion

The shift of Nino 3 SST power density towards shorter periods suggests more frequent El Nino events in future climate. Cai *et al.*^17^ has presented climate modelling evidence for a doubling in the occurrences of El Nino events in the future in response to greenhouse warming. The increase in frequency of El Nino events including extreme events could be related to diminished or reversed meridional SST gradient over the equatorial Pacific. This is associated with more occurrences of maximum SSTs in the eastern equatorial Pacific for a given SST anomaly, to increased extreme El Nino occurrences. The CMIP model simulations suggest a stable inverse relationship between El Nino and the Indian monsoon so that more frequent El Nino events could trigger more droughts over India.

The results of this study are expected to benefit the policies in place for agricultural and other managements and improve upon the existing plans for calamity mitigation arising from shifting of deficient rainfall events in shorter time band.

## Data

The set of data analysed in our work includes the time series for global SST ENSO index, Nino 3, the ISMR, and SHR7 rainfall as defined in Azad *et al.* (2010)^21^ over the historical time period 1871–2005. The raw SST data is from the NOAA Climate Prediction Center at the website http://www.cpc.ncep.noaa.gov/data/.

We basically analyse the spectral characteristics of Indian monsoon rainfall and Nino 3 SST. The ISMR is the weighted average over 36 meteorological sub-divisions and all of them do not exhibit the spectral dip in the 3–5 y period band. In fact other regions too (for example homogenous Indian monsoon (HIM ) which covers central and north western parts of India amounting to 55% of the total land area of the country) show spectral dip in this band. Since the HIM rainfall is the weighted average over 14 sub-divisions and all of them do not exhibit the spectral dip in the 3–5 y period band, therefore we selected our region of analysis to be SHR7 which spans the northern west coast to the northern part of the peninsula. This region is of particular interest as all the seven constituent sub-divisions show a spectral dip around the period of 4 y, and most of the spectral peaks in the different sub-divisions nearly coincide with each other. The SHR7 rainfall time series is obtained by area-weighted average of seven sub-divisions namely, Konkan, Madhya Maharashtra, Marathwada, Vidarbha, Telangana, Coastal Karnataka, North interior Karnataka.

We employ multi-model ensemble simulations from the world climate research program (WCRP) Coupled Model Intercomparison project 5 (CMIP5) over a period of 1871–2100 23. Annual historical simulations are analyzed and compared with observations from the Indian Institute of Tropical Meteorology (IITM) for the period (1871–2005). The future changes are analysed in 21st century (RCP8.5, 2005–2100) projected simulations. The detailed documentation of CMIP5 models can be found at: http://www.earthsystemgrid.org/search? Type=Simulation%2bMetadata.

Thus the SHR7 data are used to compare the annual rainfall from models during historical periods with observations. The analysis periods from the CMIP5 and India Meteorological Department (IMD) data sets are similar. The historical data cover a period from 1871–2005, whereas projected data cover a period from 2006–2100. This is followed by an analysis of projected rainfall using the models that have reproduced past data best.

## Method

### Spectral analysis and significance test

Estimation of the power spectral density (PSD) of a stationary random process is usually based on procedures employing the fast Fourier transform (FFT)^25^. For a discrete-time series *x*(*t*) with unit time interval (so the Nyquist frequency is 1/2) the spectral representation is a periodogram defined as


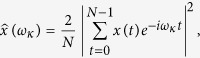


where *ω*_*k*_ = 2*Πk/N*, *N* is the sample size and *k* = 0, 1, *…*, *N/*2 is the frequency index. The value of *x*(*ω*_*k*_) is a measure of the contribution to the “energy” of *x* by the frequency *ω*_*k*_. As in our previous work, we use the Welch technique with Hamming window to estimate *x*(*ω*_*k*_). This technique uses the powerful idea of the averaged periodogram of overlapped, windowed segments of a time series, and reduces the variance associated with the standard periodogram by cutting the data into blocks and then averaging over their periodograms. To test the significance of peaks, we use the method of Torrence and Compo^29^.

## Additional Information

**How to cite this article**: Azad, S. and Rajeevan, M. Possible shift in the ENSO-Indian monsoon rainfall relationship under future global warming. *Sci. Rep.* **6**, 20145; doi: 10.1038/srep20145 (2016).

## Supplementary Material

Supplementary Information

## References

[b1] Walker G. T. & Bliss E. W. World weather V, Mem. R. Meteorol. Soc. 4, 53–84 (1932).

[b2] Rajeevan M. & McPhaden M. J. Tropical Pacific upper ocean heat content variations and Indian summer monsoon rainfall. Geophys. Res. Lett. 31, L18203, 10.1029/2004GL020631 (2004).

[b3] Sikka D. R. Some aspects of the large scale fluctuations of summer monsoon rainfall over India in relation to fluctuations in the planetary and regional scale circulation parameters. Proc. Indian Acad. Sci. Earth Planet Sci. 89, 179–195 (1980).

[b4] Kripalani R. H. & Kulkarni A. Climatic impact of El NiAo/La Niiia on the Indian monsoon: A new perspective, Weather 52(2), 39–46 (1997).

[b5] Bhattacharyya S. & Narasimha R. Regional differentiation in multidecadal connections between Indian monsoon rainfall and solar activity. J. Geophys. Res. 112, 10.1029/2006JD008353,D24103 (2007).

[b6] Gadgil S., Rajeevan M. & Francis P. A. Monsoon variability: links to major oscillations over thee quatorial Pacific and Indian oceans. Curr. Sci. 93, 182–194 (2007).

[b7] Krishna K. K., Rajagopalan B., Hoerting M., Bates G. & Cane M. Unravelling the mystery of Indian monsoon failure during El-Nino. Science. 314, 115–119 (2006). 16959975 10.1126/science.1131152

[b8] Rajeevan M. & Pai D. S. On the El Niño-Indian monsoon predictive relationships. Geophys. Res. Lett. 34, L04704, 10.1029/2006GL028916. (2007).

[b9] Saini S. & Gulati A. El Niño and Indian droughts- A scoping exercise, *Indian council for research on international economic relations*, Working paper 276 (2014).

[b10] Rajeevan M., Pai D. S., Kumar R. A. & Lal B. New statistical models for long-range forecasting of southwest monsoon rainfall over India. Clim. Dyn. 28(7–8), 813–828 (2006).

[b11] Webster P. J. & Yang S. Monsoon and ENSO: Selectively interactive systems. Q. J. R. Meteorol. Soc. 118, 877–926 (1992).

[b12] IPCC, Synthesis Report. Intergovernmental Panel on Climate Change, WMO and UNEP, Geneva, 2007.

[b13] Menon A., Levermann A., Schewe J., Lehmann J. & Frieler K. Consistent increase in Indian monsoon rainfall and its variability across CMIP-5 models. Earth. Sys. Dyn. 4, 287–300 (2013).

[b14] Sharmila S., Joseph S., Sahai A. K., Abhilash S. & Chattopadhyay R. Future projection of Indian summer monsoon variability under climate change scenario: An assessment from CMIP5 climate models. Global and Planetary Change. 124, 62–78 (2015).

[b15] Giorgi F. *et al.* Higher hydroclimatic intensity with global warming. J. Clim. 24, 5309–5324 (2011).

[b16] Singh D. *et al.* Observed changes in extreme wet and dry spells during the South Asian summer monsoon season *Nature* Clim. Change 4, 456–461 (2014).

[b17] Cai W. *et al.* Increasing frequency of extreme El Niño events due to greenhouse warming. Nature Clim. Change 4, 111–116 (2014).

[b18] Asrit K., Kumar R. & Kumar K. K. ENSO-Monsoon relationships in a greenhouse warning scenario, Geophy. Res. Lett. 28(9), 1727–1730 (2001).

[b19] Kumar K. K., Rajagopalan B. & Cane M. A. On the weakening relationship between the Indian monsoon and ENSO. Science 284, 2156–2159 (1999). 10381876 10.1126/science.284.5423.2156

[b20] Gadgil S. & Gadgil S. The Indian monsoon, GDP and agriculture. Economic and Political Weekly. 41(47), 4887–4895 (2006).

[b21] Azad S. & Vignesh T. & Narasimha R. Periodicities in Indian monsoon rainfall over spectrally homogeneous regions. Int. J. Climatol. 30, 2289–2298 (2010).

[b22] Narasimha R. & Bhattacharyya S. A wavelet cross-spectral analysis of solar–ENSO– rainfall connections in the Indian monsoons. Appl. Comput. Harmon. Anal. 28, 285–295 (2010).

[b23] Power S., Delage F., Chung C., Kociuba G. & Keay K. Robust twenty-first-century projections of El Niño and related precipitation variability. Nature 502, 541–545 (2013). 24121439 10.1038/nature12580

[b24] Taylor K. E., Stouffer R. J. & Meehl G. A. An Overview of CMIP5 and the Experiment Design. Bull. Amer. Meteor. Soc. 93, 485–498 (2012).

[b25] Welch P. D. The Use of Fast Fourier Transform for the Estimation of Power Spectra: A Method Based on Time Averaging Over Short, Modi_ed Periodograms. *IEEE Trans. Audio Electroacoust*. AU-15, 70–73 (1967).

[b26] Azad S. & Narasimha R. A wavelet based significance test for periodicities in Indian monsoon rainfall. International Journal of Wavelets, Multi-resolution and Information Processing. 6(2), 291–304 (2008).

[b27] Kug J. S., An S. I., Ham Y. G. & Kang I. S. Changes in El Niño and La Niña teleconnections over North Pacific–America in the global warming simulations. Theor. Appl. Climatol. 100, 275–282 (2010).

[b28] Stuecker M. F., Timmermann A. Jin F.-F., McGregor S. & Ren H.-L. A combination mode of the annual cycle and the El Nino/Southern Oscillation. Nat. Geosci. 6, 540–544 (2013).

[b29] Torrence C. & Compo G. C. A practical guide to wavelet analysis. Bull. Amer. Met. Soc. 79(1), 61–78 (1998).

