# Supplementary material for: Possible shift in the ENSO-Indian monsoon rainfall relationship under future global warming
Source: Sci Rep. 2016 Feb 3;6:20145. doi: 10.1038/srep20145 (PMC4738276; doi:10.1038/srep20145)
Supplement: Supplementary Information [file srep20145-s1.doc]

**Possible shift in the ENSO-Indian monsoon relationship under future global warming**

^*^Sarita Azad, ^a^M. Rajeevan

* Indian Institute of Technology Mandi,

Mandi 75001 Himachal Pradesh, India

[sarita@iitmandi.ac.in](mailto:sarita@iitmandi.ac.in)

^a^ Indian Institute of Tropical Meteorology

Dr Homi Bhabha Road, Pashan,

Pune- 411 008


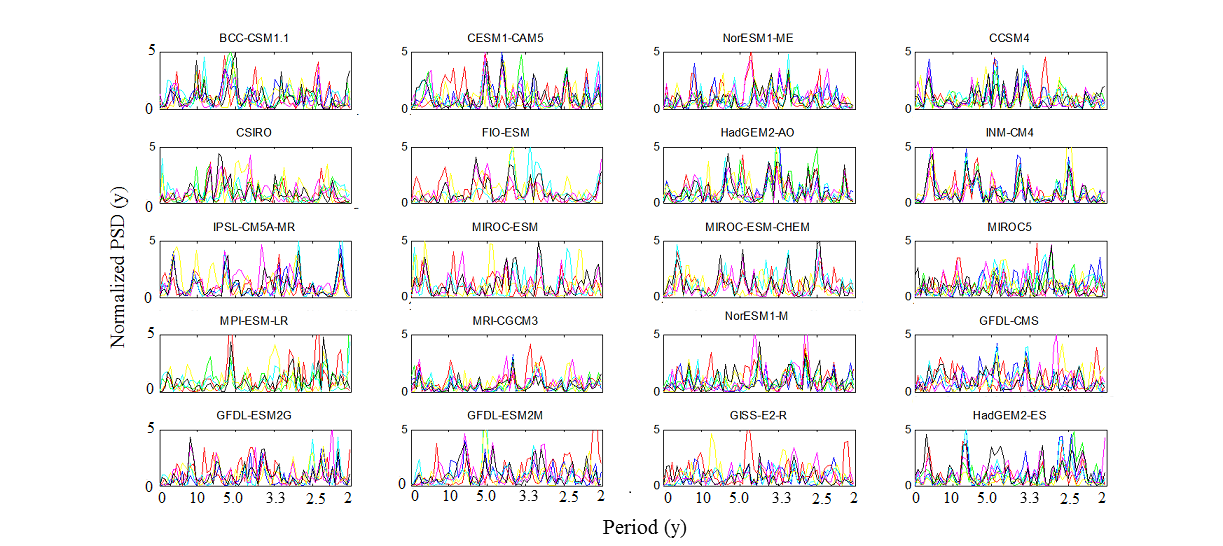


Fig. S1 Estimated PSD of seven sub-divisions of 20 models (time period 1871-2005)


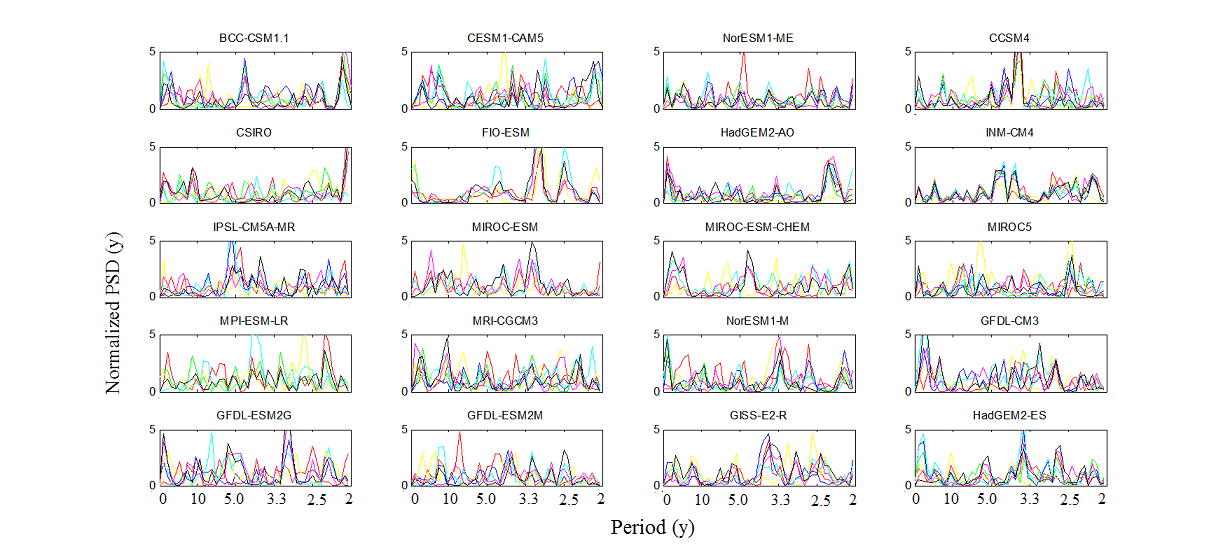


Fig. S2 Estimated PSD of seven sub-divisions of 20 projected models (time period 2006-2100)


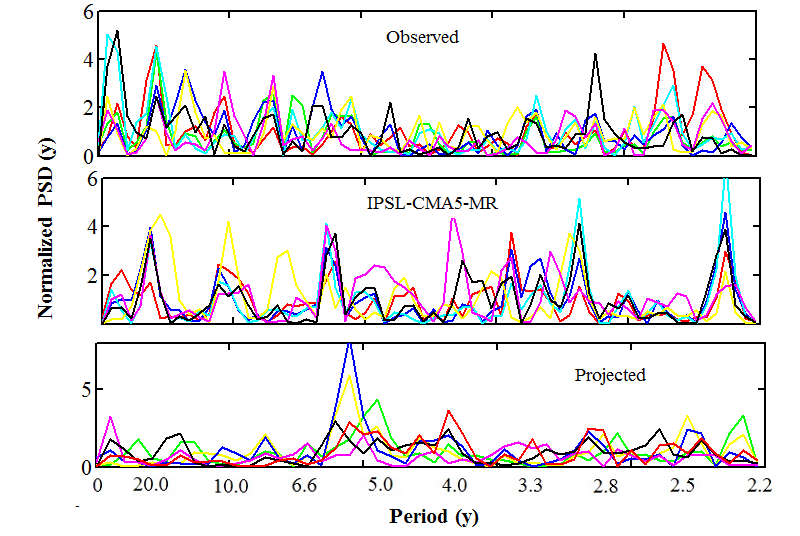


Fig. S3 Estimated PSD of seven sub-divisions of (a) observed; (b) IPSL-CM5A-MR model; and (c) projected.
